# Long-Term Care for Tracheotomised Patients With or Without Invasive Ventilation. Lessons Learned from a Scoping Review of International Concepts

**DOI:** 10.5334/ijic.5429

**Published:** 2020-07-17

**Authors:** Susanne Stark, Michael Ewers

**Affiliations:** 1 Charité – Universitätsmedizin Berlin, corporate member of Freie Universität Berlin, Humboldt-Universität zu Berlin, and Berlin Institute of Health, Institute of Health and Nursing Science, Berlin, DE

**Keywords:** complex healthcare needs, long-term tracheostomy, mechanical ventilation, integrated care, care coordination, healthcare concepts, scoping review

## Abstract

**Introduction:**

Patients with long-term tracheostomies, with or without mechanical ventilation have complex and comprehensive healthcare needs. The number of patients is increasing internationally. Evidence suggests poor healthcare quality and outcomes, especially in Germany. Against this background, we searched for international concepts tailoring healthcare to these special needs, their key characteristics and results from their evaluations.

**Methods:**

A scoping review was performed in 2018 based on a systematic search of scientific databases and grey literature without restrictions to publication type. Key information was charted and thematically analysed based on the taxonomy of integrated care. Evaluations were analysed descriptively.

**Results:**

Seventy-nine publications related to 25 programmes from five countries were included. Healthcare concepts are usually regionally adapted and tertiary sector-based with a cross-sectoral approach. Care coordination responsibility is usually assigned to advanced nurse practitioners, embedded in multi-professional programme teams. Interventions consist of specialised needs-based clinical services combined with care coordination, homecare support and education. Evaluation of concepts is scarce, but existing results indicate beneficial effects on patient-related outcomes, care coordination, healthcare utilisation and costs.

**Conclusions:**

The concepts available in the literature are often poorly described and rarely evaluated. Research is needed on their impact on healthcare quality and outcomes. However, several key characteristics were identified, which should be considered when developing and implementing integrated and needs-based approaches for the patient group in Germany and beyond.

## Introduction

Patients with long-term tracheostomies, with or without mechanical ventilation, belong to a special patient group with complex and comprehensive healthcare needs. This is due to severe, often multiple chronic conditions and functional limitations [1,2]. Demographic and epidemiological changes aligned with advances in medical technology and the medicalisation of healthcare are leading to a steady increase of this and other patient groups with a long-term dependency on healthcare technology [1,3,4]. The complexity of their needs often requires the support of ongoing multifaceted, highly specialised professional health and social services spanning different sectors, settings and care levels [5,6]. The services provided should be tailored to those needs and emphasize the integration and coordination of high quality, safe and timely care from a continuity perspective to provide the right care, in the right place, at the right time [6,7,8]. This often proves challenging – as the following outline of the German and international situation highlights.

An estimated 15,000 to 30,000 patients with long-term tracheostomies – mostly invasively ventilated – live in German community settings [9]. Although reliable data are missing [10,11], the patient group is expected to grow in line with international epidemiological trends [9]. However, strategies to adequately address the complex and increasing demands of their specialised healthcare are lacking. Concepts that meet the patients’ complex needs are yet insufficiently described, evaluated or disseminated. Needs-orientation, quality, continuity and integration of specialised healthcare is reasonably questioned due to missing regulations, service fragmentation, often poorly qualified professionals and a mainly economically-driven rather than needs-oriented service development [12,13]. For example, premature hospital discharges, inadequate weaning and rehabilitation opportunities or a lack of care coordination are criticised [12].

Although a German guideline on home mechanical ventilation provides recommendations on necessary healthcare structures, professional qualifications, discharge procedures, follow-up and healthcare monitoring [14,15]. However, they are not mandatory, mainly focus the acute care setting [12] and lack overarching healthcare coordination and integration strategies. In addition, single local initiatives to improved specialised healthcare usually lack information on their elements, services and outcomes [11].

These challenges are not unique to the German context. Service fragmentation, regional healthcare disparities, access barriers and poor healthcare quality, as well as inconsistent professional expertise seem to affect adequate specialised healthcare in various other countries [16,17,18,19,20,21,22,23,24,25]. However, even internationally, little is known about strategies to adequately meet the complex needs of tracheotomised patients with or without mechanical ventilation throughout the healthcare continuum.

Originating from the German situation, this scoping review asks about specific approaches or concepts reported in the international literature that are tailored to improve specialised healthcare. The aim is to map and understand the key features that characterise such approaches regarding the range, elements, processes and outcomes of the services provided. Based on this overview, conclusions will be drawn on the future development of sound concepts and healthcare strategies. These implications are particularly relevant for Germany, but also other countries facing the challenges outlined above. The results should, therefore, contribute to the international discourse on needs-based healthcare for the patient group, with special focus on appropriate cross-sectoral care, overall healthcare goals, patient pathways, provider qualification and responsibilities to avoid over- or undersupply and inappropriate healthcare.

## Theory and Methods

A scoping review [26,27] was conducted corresponding to the explorative nature of the research. According to the aim of this method to systematically search, collate and map existing knowledge on a broad field of conceptual practice, research, evidence and gaps [27], we summarised and mapped the so far scarcely researched field of healthcare approaches relevant to the patient group. The methodological approach, guided by the PRISMA reporting checklist for scoping reviews [28], followed five proposed steps of scoping studies: (a) identifying the research question, (b) identifying relevant studies, (c) study selection, (d) data charting, and (e) data collation and thematic analysis [27].

### Systematic search strategy

Search terms and strategies to identify relevant concepts and related research were developed and piloted with an initial CINAHL and PubMed (incl. MEDLINE) search, covering relevant medical, nursing and life science publications. The records obtained were screened and discussed in the research team. The search strategy was subsequently refined. Since the pilot search revealed a small body of scientific literature and to achieve comprehensiveness, the final CINAHL and PubMed search of the English literature, conducted in April 2018, was particularly extended by grey literature search (google scholar), snowball search of reference lists from all full texts included and hand-search (google) of online sources related to concepts identified. Authors were additionally contacted personally (via mail, n = 5) whenever published information was insufficient for the analysis. The search terms systematically combined using the SPICE scheme [29] are shown in Table 1. Evaluation and control criteria were not decisive for inclusion or exclusion and, thus, were not specified. The search was limited to the earliest publication date of 2000, assuming that previous publications would not adequately reflect current healthcare practice, since medical innovations, healthcare regulations, paradigms and with them, specialised services changed dynamically over the years in Germany and internationally.

**Table 1:** Search terms and combination.

Domain of the SPICE scheme [29]	Search terms

Setting	community/home/outpatient/domicil*/resident*/long-term
Perspective (Patient group)	tracheostom*/tracheotom*/mechanic*/artific*/invasiv* AND respirat*/ventilat*
Intervention	inter-/multi-disciplinary, inter-/multi-professional, cross-/inter-/multi-sectoral AND care cooperat*/coordinat*/team ANDconcept/project/plan/pathway/support/service/complex intervention, case/care management, managed/integrated/continu* care
Comparison	Not specified
Evaluation	Not specified

Inclusion criteria were defined as follows. The concepts identified must a) currently be operated and b) address at least one of the following patient groups: children/adolescents or adults with complex healthcare needs due to tracheotomy with or without mechanical ventilation. Concepts must further c) address the community setting, i.e. a (nursing) home or equivalent places of residence, affect at least two different healthcare sectors and d) be multi-professionally designed, addressing the complexity of patients’ needs. No restrictions were defined for publication types and quality. A formal quality assessment of the literature was refrained from since we aimed to explore and describe the extent, range and nature of knowledge and the literature, rather than to evaluate the effectiveness of the concepts identified. However, study designs are reported and discussed.

Records were excluded if the concepts described a) do not address the patient group or b) the community settings, c) focus solely on single or mono-professional interventions (e.g. clinical, medication, medical supply or discharge management) or d) if relevant data were not available by hand search or request. Title, abstract, and full-text screening against inclusion and exclusion criteria was conducted by two researchers independently. Inconsistencies were discussed and consented within the research team.

### Publication selection, data extraction and analysis

After duplicates were removed, data from eligible publications were extracted according to a deductive approach and categorized based on a charting form developed from the taxonomy of integrated care. According to this taxonomy, healthcare integration comprises clinical, professional, organisational, systemic and functional dimensions [6,30,31,32]. Approaches on integration aim to support continuity, coordination and collaboration of healthcare and have the potential to enhance the quality and outcomes of healthcare [30,33,34]. The taxonomy is, therefore, eligible to comprehensively describe key characteristics of concepts that aim to address the patient group’s complexity of needs. The charting form contains formal attributes of each concept (name, country, region) and the following descriptive dimensions: reported model of care, population (target patient group, territory covered), organisational characteristics (sectors involved, leading sector), professional characteristics (professions involved, operational leadership), process characteristics (key interventions, availability of standards), and management, funding and policy information. If concepts were evaluated, study characteristics (design, population, indicators, and measures), primary outcomes, the scope and extent of research activities were extracted. One reviewer (ST) drew responsible for initial data extraction, a second reviewer (ME) conducted an independent extraction for a random sample of publications (n = 6). Inconsistencies were discussed until agreement was reached. The charted data were summarised thematically by collating similar aspects and retaining differences within the above-mentioned dimensions. The results were tabulated and narratively reported.

## Results

**Figure 1:**
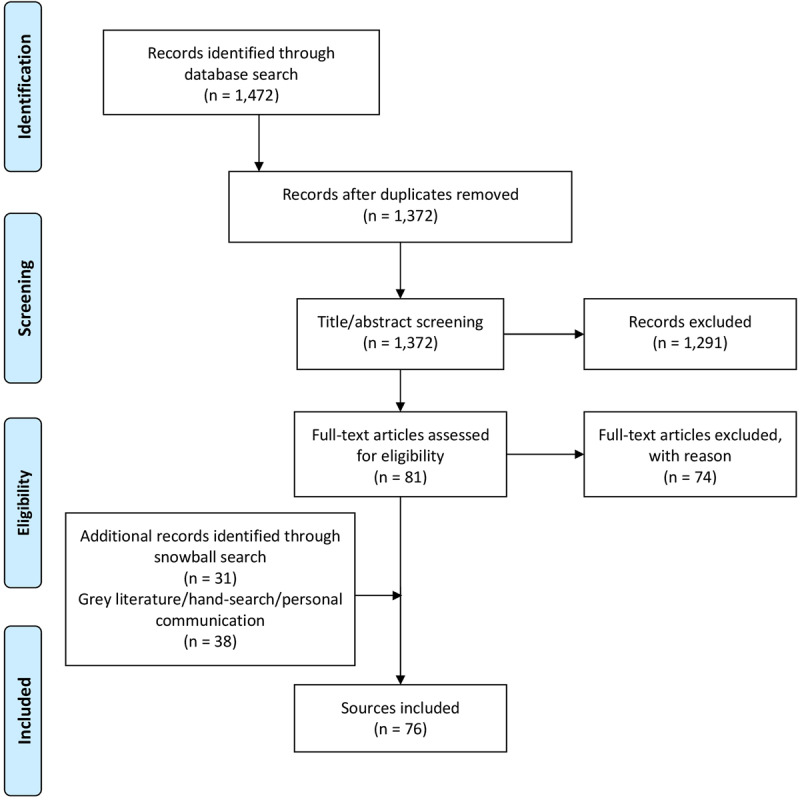
PRISMA flow chart of the systematic literature search.

The database search yielded 1,472 records. After removal of duplicates, screening for eligibility, grey literature/hand- search and personal requests, 76 sources representing 25 single concepts were finally included in the review. Please see Appendix 1 for the list of reviewed references. Of those records, 57 are descriptive concept reports and 19 represent research papers. Figure 1 illustrates the literature selection process. The concepts included originate from five countries: five Australian, six Canadian, 13 US-American and one concept each from France and Spain were reviewed (Table 2). The number of publications per concept ranges from one (n = 4) to nine (n = 1), with a mean of three publications (Table 3). The key characteristics of concepts, interventions and evaluations are reported in the sections below and summarised in Tables 3 and 4 (see Appendix 2).

**Table 2:** Concepts identified.

No.	Country	Region	Programme title	Model of care reported

1	AUS	Victoria	Complex Care Hub	NS
2	AUS	Queensland, New South Wales	Connected Care Program	Shared Care
3	AUS	Victoria	Victorian Respiratory Support Service (VRSS)	NS
4	AUS	Victoria, Tasmania, New South Wales	Tracheostomy Review and Management Service (TRAMS)	NS
S	CA	Ontario	Champlain Complex Care Program	Medical Home
6	CA	Toronto, Southern Ontario	Complex Care Program	Medical Home/Shared Care
7	CA	British Columbia	Provincial Respiratory Outreach Program (PROP)	NS
8	CA	Quebec	Quebec National Program for Home Ventilatory Assistance (NPHVA)	NS
9	CA	South West Ontario	Chronic Mechanical Ventilation Program	Systems Model of Integrated Care
10	CA	Toronto	Long-Term Ventilation Centre of Excellence (LTVCOE)	NS
11	ES	Barcelona	Plataforma de Respuesta Integral a Niños Crónicos con Elevada dependencia (PRINCEP)	Case Management
12	FR	France	Fédération Association Nationale pour le Traitement à Domicile de l’ Insuffisance Respiratoire Chronique (ANTADIR)	Statutory
13	USA	Michigan	Assisted Ventilation Clinic (AVC)	Case Management
14	USA	North Carolina	Child Health Accountable Care Collaborative (CHACC)	Medical Home
15	USA	Illinois	Continuity of Care Program lowa (COC)	Medical Home
16	USA	Michigan	Pediatric Home Ventilator Program	NS
17	USA	Pennsylvania	Technology Assisted Children’s Home Program (TACHP)	NS
18	USA	Pennsylvania	Home Ventilator Program, Children’s Hospital of Philadelphia (CHOP)	NS
19	USA	Wisconsin, Illinois	Special Needs Program (SNP)	Medical Home
20	USA	Midwest	Tracheostomy and Home Ventilator Program	NS
21	USA	New England	Critical Care, Anesthesia, Perioperative Extension (CAPE) and Home Ventilator Program	supplemented Medical Home
22	USA	Massachusetts	Pediatric Alliance for Coordinated Care (PACC)	Medical Home
23	USA	Indiana	Home Ventilation Program	NS
24	USA	Maryland	Rare and Expensive Case Management (REM) Program	Case Management
25	USA	Minnesota	Special Needs Program (SNP) University of Minnesota (U of M)	Shared Care, Medical Home Centre

AUS – Australia; CA – Canada; FR – France; ES – Spain; USA – United States of America.

**Table 3:**
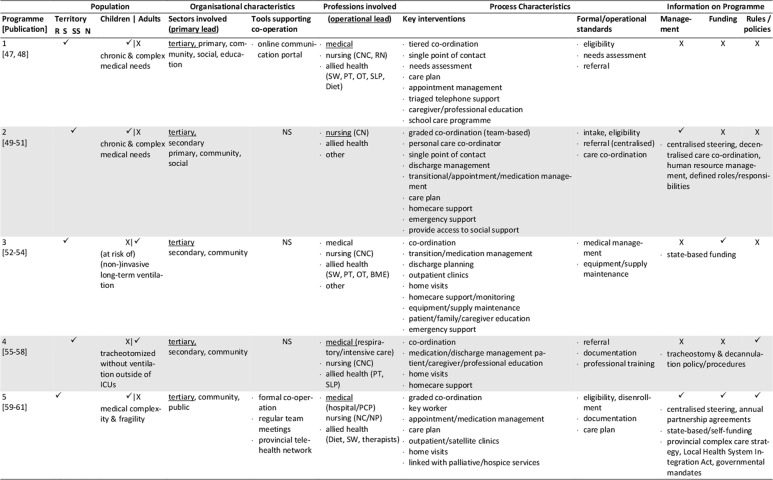
Concept characteristics.

**Figure:**
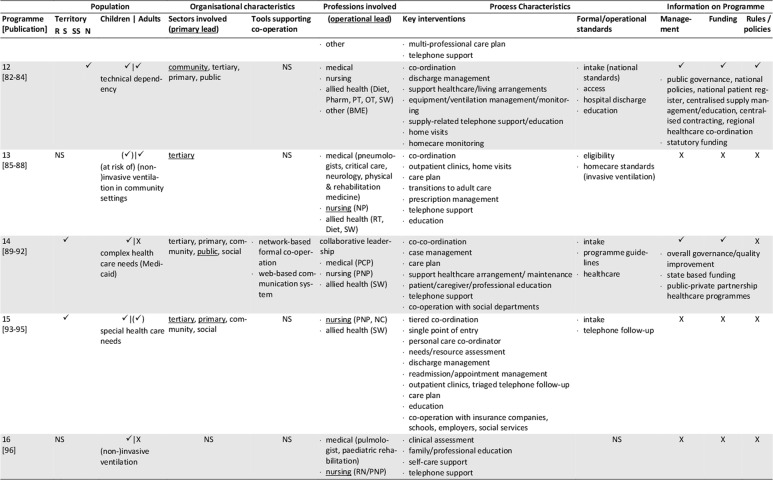


**Figure:**
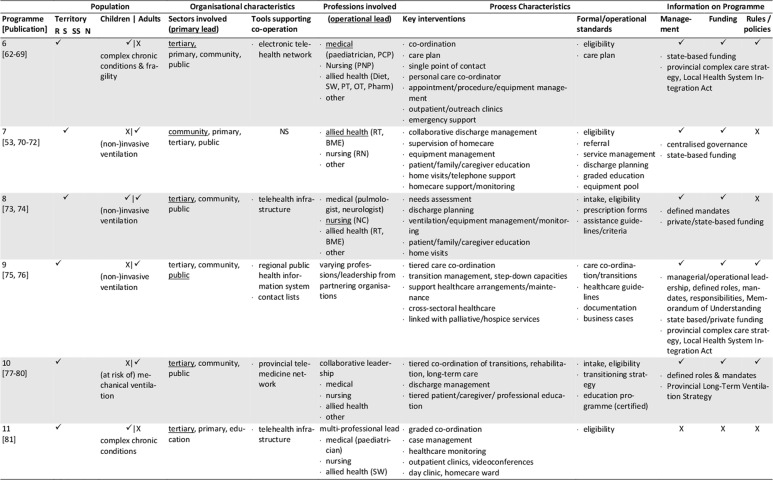


**Figure:**
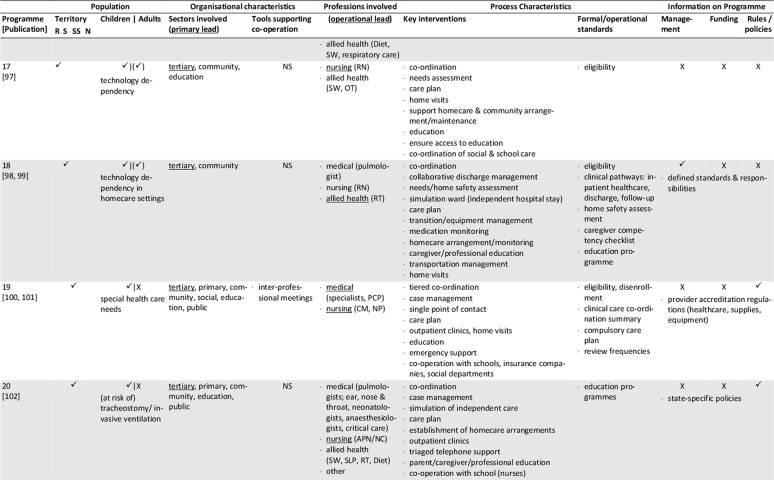


**Figure:**
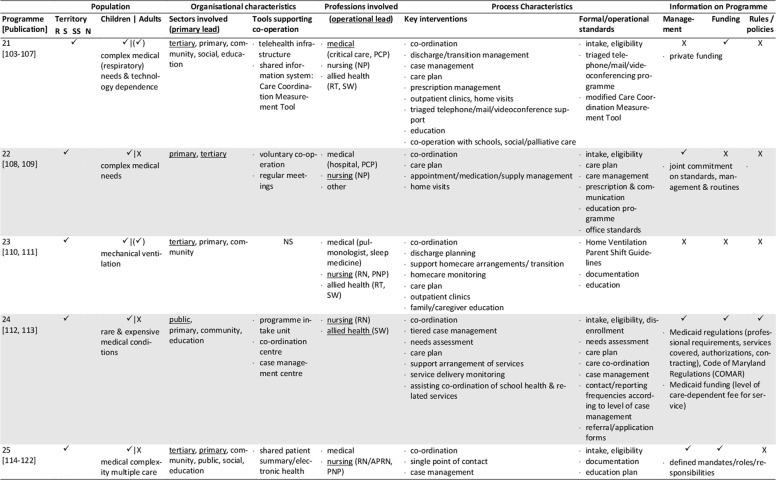


**Figure:**
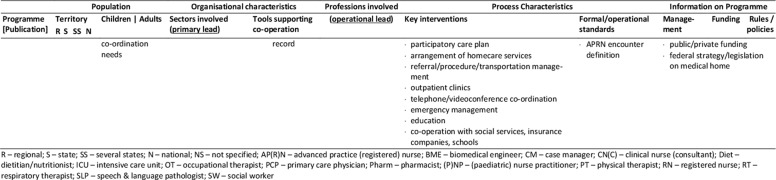


### Reported models of care

The majority of the concepts reviewed are built on individual approaches (Table 3): six concepts (24%) are assigned to the medical home, three (12%) apply case management, one employs a shared care approach, and two concepts combine the medical home and shared care. One concept each applies an integrated care concept (No. 9, [76]), or a national approach (No. 12, [84]). No specific model of care is reported for the remaining programmes (n = 11, 44%).

### Population: target patient group and catchment area

Most concepts are tailored to children and adolescents (n = 17, 72%), a quarter addresses adult patients (n = 6, 24%) and few programmes are aimed at all age groups (n = 2, 8%). The main conditions addressed are complex medical conditions/medical complexity (n = 10, 40%), including tracheotomised patients with or without mechanical ventilation. Programmes with a narrower medical scope focus technology dependency (n = 4; 16%), (non-)invasive mechanical ventilation (n = 7; 28%), tracheostomy (n = 2) or invasive ventilation only (n = 1). Specific intake criteria are reported for 18 concepts (72%), of which half (n = 10) rely on complexity or fragility criteria.

Most of the programmes cover a defined catchment area, commonly a state/provincial (n = 11; 44%) or a regional/local level (n = 6; 24%). Other programmes cover territories beyond the state-level (n = 5; 20%), with the French model (No. 12) being the only one with a national scope (missing information: n = 2). Some programmes are also located in similar regions, partially covering similar territories or patient groups (Table 2). Concepts with larger catchment areas partly operate satellite or outreach clinics (No. 3, 5, 6) or local health networks (No. 2, 12) to ensure specialised service provision near the patient’s place of residence.

### Sectors and organisations involved in healthcare and care coordination

Most programmes are located at specialised departments of tertiary hospitals that have responsibility (primary lead) for care coordination (n = 16, 64%, Table 3). Fewer programmes share care coordination responsibilities between both, the tertiary and primary care sector (n = 3, 12%), or entirely allocate responsibility to the public (n = 3, 12%) or community sector (n = 2, 8%). About half of the concepts utilise a cross-sectoral approach covering specialised tertiary, primary and community care (n = 13, 52%), or at least either the tertiary and community (n = 7, 36%) or the tertiary and primary sector (n = 2, 8%). Few concepts also integrate the social (n = 7, 28%) and public sector (n = 9, 36%) or the education system (n = 8, 32%).

Information on how concepts design inter-organisational collaboration within and across sectors is rare, especially regarding collaborations beyond the health system (n = 17, 68%). Formal agreements or contracts on professional/organisational responsibilities/requirements or standards are reported for five programmes (20%). (Informal) networking is common in most concepts (n = 18, 72%). Information on the types and extent of inter-organisational relationships is generally lacking. Most programmes providing information on tools supporting co-operation and coordination (n = 12, 48%), use facility-specific or public digital information systems (n = 9, 36%), single e-health instruments (e.g. shared care plans, health records, documentation templates; n = 2, 8%) or regular face-to-face meetings (12%).

### Professions involved, professional leadership

All programmes have multi-professional teams, usually affiliated to the programme hosting institution. The reported staff composition is heterogeneous. At least specialised experts from medicine, nursing and social work are involved (missing information: n = 2). Primary care is also a common part of most concepts, usually realised through direct connection to the primary care physician (n = 15, 60%). Contrarily, allied health professions, consisting of occupational, physical and speech and language therapists (n = 9, 36%), dietitians (n = 7, 28%), respiratory therapists (n = 9, 36%), biomedical engineers (n = 4, 16%) or pharmacists (n = 3, 12%) are not regularly involved.

The operational lead of programmes is predominantly held by advanced nursing professionals (n = 10, 40%), or in co-operation between nursing and other professionals (n = 6, 24%). Medical (n = 4, 16%), respiratory therapist (n = 1, 4%), collaborative (n = 2, 8%) or alternating (n = 1, 4%) coordination responsibility occurs less frequently (missing information: n = 2). If primary care physicians assume coordination responsibility, they are particularly in charge of primary care coordination. The involvement of dedicated case managers (n = 6, 24%) or additional coordination personnel (e.g. patient information coordinator, technical coordinator, allied health coordinator) is rarely reported. Transparency regarding the specific staff training and qualifications is low, except for physicians and respiratory therapists.

### Key interventions and processes

The typical programme interventions and processes show considerable heterogeneity, but also similarities. Usually, interventions are provided on an individual basis (n = 16, 64%). About one third (n = 9, 36%) of the programmes are built upon graded approaches that systematically navigate patients to specific services according to their needs. Patients with maximum complexity receive intensive care management and dense follow-up, which is tiered down with decreasing complexity.

Programme intake is managed variably, predominantly starting during hospital stays (n = 22, 88%). Access to concepts and communication processes with patients and/or informal caregivers is managed either by case managers (n = 7, 28%), single points of contact (n = 7, 28%) or personal care coordinators (n = 4, 16%; missing information: n = 9, 36%).

The reported **care coordination** interventions usually comprise inpatient (including discharge), outpatient and home healthcare (n = 14, 56%), or parts of the healthcare continuum (n = 8, 32%; missing information: n = 2). Some concepts additionally coordinate transitions to adulthood (n = 2, 8%) or palliative/hospice services (n = 3, 12%). Coordination beyond the health system is less typical. If provided, those services comprise collaboration with social services (n = 7, 28%), insurance companies (n = 1, 4%), or schools/employers (n = 9, 36%).

Typical coordination tasks of the programmes concern at least, medical, nursing, social, medication and nutrition aspects. Other common components of care coordination are prescription and/or equipment management (n = 12, 48%), and/or appointment management (n = 6, 24%). Some concepts further coordinate or assist with emergency support (n = 5, 20%), resource or home safety assessments (n = 2, 8%) or transportation management (n = 1, 4%).

The primary tools applied to guide and support care coordination are care plans (n = 17, 68%) and/or needs assessments (n = 6, 24%), initially prepared prior to hospital discharge or at the time of programme enrolment, and regularly updated. Further tools are rarely reported. The information available varies and mostly refers to formal or procedural standards, especially regarding documentation (n = 9, 36%), care plans (n = 4, 16%) or needs assessments (n = 3, 12%), training and educational programmes (n = 9, 36%), the referral, medication, equipment and/or ventilation management demands (n = 7, 28%), or care coordination (n = 6, 24%).

**Inpatient care** coordination often directly includes specialised acute services (n = 14, 56%, e.g. respiratory monitoring, decannulation, weaning or initiation of ventilation), or refers to the co-operation with inpatient care coordinators (n = 9, 36%). Additionally, the preparation, coordination and conduct of discharges (n = 11, 44%) or transitions (n = 8, 32%) are typical coordination tasks, which are sometimes prepared by step-down unit (No. 9) or independent care ward stays (No. 18, 20).

Beyond inpatient care, **regular multi-professional follow-up** for ongoing needs-based support is a key feature of most programmes. Typically, they are conducted as outpatient clinic visits (n = 11, 44%) scheduled at the programme host or outreach clinics, as home visits (n = 13, 52%) or telehealth appointments (n = 4, 16%). The reported frequencies vary and are either scheduled needs-based or fixed (e.g. monthly, annual). Follow-up commonly covers specialised examinations, needs assessments, care plan updates and the monitoring and/or coordination of home care arrangements. Individual programmes aim to schedule all procedures into one single appointment (No. 13, 20, 25) to bundle specialised care and reduce patient/family efforts.

Another key element of most programmes is **homecare coordination.** However, the levels of monitoring and support differ, with direct participation in home healthcare being rare (n = 1). **Primary care** coordination is fundamental to several concepts, and is often accounted for by the primary care physician (n = 15, 60%) or shared with programme team members in charge. Some providers support the establishment and maintenance of care arrangements and contacts with providers (n = 10, 40%). Services may also include the setup, maintenance and/or monitoring of technical equipment and homecare (n = 7, 28%). Homecare is usually coordinated through home visits and/or telehealth support by multi-professional teams or single professionals. Telehealth via telephone (n = 9, 36%) or videoconferences (n = 3, 12%) is partly supported by triage systems (n = 6, 24%). Some concepts offer 24/7 support services, others during office hours, often complemented by after-hour backup. Contact frequencies vary from needs-based to fixed appointments (e.g. monthly, annual).

An additional obligatory component of most concepts (n = 18, 72%) is (structured) patient, caregiver and/or provider **education and training**, which usually starts during discharge or programme enrolment and is offered continuously. The interventions aim to prepare participants for homecare by providing information about, for example, health conditions and treatments, homecare safety, the handling of medical aids and devices, troubleshooting or emergency procedures.

### Programme management and funding; underlying rules and policies

Information on programme management is missing for nearly half of the concepts (n = 12, 48%) or incomplete. The reported management types are public (n = 4), medical (n = 2), institutional (n = 1) or multi-organisational (n = 2). Programmes with public involvement are reported to have specific healthcare and care coordination mandates.

The reported funding sources of twelve programmes (48%) are predominantly public (n = 8), sometimes supplemented by private (organisational) input (n = 3) and patient co-payments (n = 1). Exclusive private funding is exceptional (n = 1).

Underlying public policies or rules guiding programmatic approaches are partially reported (n = 10, 40%). They either refer to a state (n = 9, 36%) or the national level (No. 12) and concern programme structures, standards, integration strategies, or service/professional regulations.

### Evaluation

A total of 19 evaluations relating to twelve concepts were identified (48%, Table 4, see Appendix 2). Two concepts (No. 6, 25) were evaluated with several studies. Information on internal or external evaluation approaches is generally lacking. The evaluation designs utilised are mostly non-experimental. Two studies each used a mixed-method or qualitative approach. Study sample sizes (patients and/or caregivers) range from a maximum of 50 subjects (n = 9), over 51–150 participants (n = 4) up to 158–1,558 participants (n = 5).

Most evaluations (n = 15, 79%) focus on healthcare outcome indicators (e.g. resource utilisation [n = 10], adverse events [n = 2]), patient/family reported outcomes (e.g. experience and/or satisfaction with healthcare or care coordination [n = 6], perceived value of interventions [n = 1], health-related quality of life [n = 3]) or economic indicators (e.g. health and/or social care costs [n = 7]). Few indicators refer to outputs (e.g. intervention characteristics [n = 4] and results [n = 1]). Process evaluations (n = 5, 26%) examine barriers, facilitators and values of collaboration or interventions. The measures utilised vary considerably.

The evaluation results indicate that concepts and interventions could contribute to decreased (un-)planned hospital and/or emergency department admissions [49, 59, 100, 104, 105, 108, 116, 119], hospital length of stay [49, 56, 59, 63, 64, 95, 100] or programme service utilisation [49, 105]. This shift towards less inpatient utilisation is reportedly associated with lower overall healthcare costs [56, 64, 95, 100, 108, 112]. In comparison, studies also show an increased utilisation of outpatient services [49, 59, 63, 100]. Further, patients and families seem to be satisfied with care continuity and coordination [63, 64, 66, 104, 105, 108, 119, 100], with service integration [64] or support [66, 119]. However, outcomes are not consistent between evaluations, and the methodological heterogeneity complicates the comparability of results.

## Discussion

### Lessons learned

Patients with long-term tracheotomies, with or without mechanical ventilation, usually require ongoing multifaceted, highly specialised professional health and social services spanning different sectors, settings and care levels [5,6]. Since German approaches to address this complexity are widely missing and poorly reported [11], this scoping review explored and summarised the international research landscape on healthcare concepts that aim to provide the right care at the right time in the right place for these and comparable patients. The resulting overview should contribute responding to challenges, which are typical but not unique to the German situation. The implications outlined below are, therefore, of particular international relevance.

The systematic search of the published literature revealed a diverse set of concepts established in 5 countries that comprise specialised services across the healthcare continuum. However, the available information is heterogeneous, resulting in a generally poor reporting comprehensiveness. For example, only about 50% of concepts reported any conceptual approach. Information on inter-organisational collaboration, programme management, funding and regulation, intervention characteristics, or professional qualification is usually insufficient. Moreover, merely half of the concepts have undergone any evaluation and the methodological heterogeneity of studies challenges a synthesis of effects. Notwithstanding, the evaluation results indicate that comprehensive approaches can positively affect resource utilisation, healthcare costs, provider, patient and informal caregiver satisfaction, as well as the perceived care continuity and co-operation. These findings, together with the identified concept key elements, reflect the existing knowledge on preconditions and facilitators to needs-based healthcare for vulnerable and complex patient groups [ex. 6,34,35]. They may, thus, guide future (scientific) discourses and the development of sound concepts and strategies for this special patient group.

#### Conceptual key elements and characteristics

1) Application of individual conceptual frameworks tailored to a defined patient group

The most frequently reported care models are based on individual approaches (e.g. medical home, case management) that adapt interventions according to the complexity of patient’s needs. In addition, the concepts are usually sub-specialised on tracheostomized patients with or without mechanical ventilation, but generally focus on a broader population of either children/adolescents or adults with medical complexity or technology dependency.

The value of individual approaches has already been evaluated for high-need populations [8,36,37,38,39,40] and should, therefore, be taken into account. Targeting broad patient groups may reflect similarities of demands on healthcare and care coordination between different patient groups with complex needs [1,41]. In this case, resources, professional and coordination expertise could be tied up and healthcare provision and coordination could be adapted to specific subpopulations according to their needs. Evidence on the (dis-)advantages of focusing broad rather than narrow patient groups is currently lacking. However, according to existing recommendations for improving health care, approaches should carefully define the target patient group, taking into account the different needs of subgroups [7,35].

2) Regional adaption

Most of the concepts operate in small geographic regions (regional or state) and are tailored to regional characteristics, healthcare structures and services. Concepts with wider catchment areas facilitate access to and coordination of specialised services through decentralised services, such as outreach teams, satellites, local centres, telehealth interventions or home visits. Despite the limited evidence on the feasibility, benefits and effectiveness of the single approaches, it has been shown that regional adaption supports the compensation of lacking local expertise, eases access to appropriate services and improves service efficiency [34]. Therefore, future concepts should carefully be adapted to the specific regional context they are operated within.

3) Hospital-based cross-sectoral approaches

The concepts typically follow a holistic approach aiming at care continuity and integration. To achieve this, different sectors and settings are linked together. Typically implemented at highly specialised departments of tertiary hospitals, expertise is provided covering inpatient services, outpatient follow-up, home and primary care. Some concepts even reach beyond the healthcare sector and include social and educational aspects. Inter-organisational collaboration is usually built upon (in-)formal networks and supported by digital instruments.

These approaches reflect the principles of continuing, vertically and horizontally integrated services [ex. 6,7,30,42].

The link to tertiary care and the cross-sectoral approach allow a continued connection and monitoring of patients to specialists, who are usually not readily available in long-term care settings [16]. Although the specific co-operation, especially with non-healthcare parties, modes are poorly described in the literature examined, general evidence points to the crucial role of inter-organisational and cross-sectoral collaboration, clear governance, adequate responsibilities, support instruments and processes in healthcare, especially for vulnerable patient groups [6,7,42]. This should, therefore, be considered for the future development and implementation of specific concepts.

4) Multi-professional teams with nurse coordinators

All concepts are built upon multi-professional team-based approaches. Those specialised teams act within and beyond tertiary care and provide professionals, patients and caregivers with clinical expertise and coordination. Team composition differs, but at least medical, nursing and social work experts are involved according to the patients’ complex needs [1,2]. Coordination responsibility is often assigned to advanced nurse professionals, partly in collaboration with other health professions. Coordinators and programme teams often keep the responsibility for the whole coordination process. The primary care physician’s crucial role in healthcare is usually acknowledged through active co-operation and primary care (coordination) responsibilities. This collaborative approach recognises the specific competencies of all professions involved and takes account of frequently lacking expertise in non-tertiary settings [16]. It should, therefore, be carefully considered as a basic principle for the (further) development of other concepts.

5) Needs-based services combined with care coordination, homecare support and education

The concepts usually address all dimensions of healthcare across different sectors and settings, contributing to enhance care continuity and reduce fragmentation. The services include specialised, partly graded, needs-based healthcare, complemented by care coordination, homecare support and education. To what extent and how these services are provided varies considerably. Some concepts initiate services at the *inpatient setting* and typically attend hospital discharge and transitions. Most concepts organise, coordinate and conduct *regular multi-professional follow-ups*, usually held with outpatient clinic visits in varying types (face-to-face, telehealth) and frequencies. Coordination often additionally includes homecare *monitoring/coordination* and ongoing patient and family support.

Patient and caregiver *training and education* is an essential element to most concepts examined. The interventions provided aim to ensure adequate homecare, self-management and empowerment. Although feasibility and effectiveness are rarely examined, they seem to positively affect patient and family centredness, participation and empowerment. These, in turn, are crucial for high-quality healthcare [ex. 6,7]. Eligible interventions should, therefore, be an obligatory part of considerations on conceptual developments.

### Future directions and needs for action

Given the lack of systematic reporting and evaluation outlined above, stepping up research aimed at developing theoretical and empirical evidence on the feasibility, effectiveness and efficiency of special concepts is crucial. This knowledge – irrespective of its practice- or research-based evolution – would be important for the successful dissemination and adoption of appropriate healthcare concepts for this and other patient groups. In this regard, discourses on healthcare integration and coordination can provide frameworks and guidance to concept development or improvement. Reporting and evaluation guidelines for complex interventions [ex. 43,44,45] provide strategies to systematic reporting on concept development, implementation, success factors or barriers.

Based on the results of this scoping review, the following further recommendations are particularly worth considering:

- Concept reports should provide comprehensive practical, empirical and theoretical information to support learning for future initiatives. This especially applies to the underlying (theoretical or practical) assumptions, contextual demands, the target group and catchment area, and facilitators or challenges affecting concept implementation or sustainability. Evidence and conclusions on the (dis-)advantages of those characteristics and their effects should be provided.
- Significant gaps exist regarding the information on coordination interventions, goals, standards, technologies, and types and processes of co-operation. Moreover, crucial aspects of healthcare for tracheotomised patients with or without mechanical ventilation, especially *palliative and end-of-life care*, have so far been sparsely reported or even evaluated. The extent of integration of and coordination with those services should be particularly brought into discourses.
- Qualification requirements for the professionals providing specialised services should be systematically reported to enable analyses of legal or organisational standards, regulations and recommendations. On this basis, comprehensive knowledge on professional requirements essential for the provision of high-quality care could be developed.
- Telehealth approaches might be beneficial in decentralising and facilitating access to adequate interventions and expertise – not only for hard-to-reach populations in rural and remote regions. However, telehealth implementation, safety usefulness and effectiveness have so far scarcely been evaluated in the context of long-term intensive care [104, 105, 115, 121, 122] and require closer examination.
- Merely five concepts (No. 5, 6, 21, 22, 25) were evaluated concerning patient or caregiver experience and satisfaction. Given the importance of participation, patient-centredness and empowerment for high-quality healthcare [ex. 6,7], these aspects should be particularly considered in future research and practice.
- Knowledge about professional, organisational and contextual facilitators, barriers to and effects of (sustainable) concept implementation are almost absent in the approaches reviewed. However, the diffusion and replication high-quality healthcare is fundamentally affected by these factors [ex. 6,42,46]. They should, therefore, be systematically assessed and evaluated in future research.
- Above all, research should draw on the utilisation of evidence-based, consistent, sensitive and specific evaluation indicators and measures. This is all the more important as it facilitates the comparison of different approaches, their outcomes and impacts in different contexts.

### Strengths and Limitations

Methodological limitations of this scoping review result from the restriction to the English literature. This may have fostered the exclusion of concepts published in other languages. The limitation of publication dates may have excluded current concepts, not published about during the last two decades. However, the review aimed to reflect current knowledge and practices to support the *future* development of concepts. This knowledge was not expected to be found in publications before the year 2000.

Search term sensitivity or specificity, non-reporting or reporting bias may also have had limiting effects. To avoid this, the search strategy was carefully reflected on within the research team. Further information could have been obtained through the systematic inclusion of unpublished knowledge, e.g. by consultation of local experts and programme leaders. Limitations based on the search restriction to two scientific databases were expected to be minor, since both cover a wide range of medical, nursing and life sciences and the initial screening revealed a small body of literature.

The findings were collated from concepts, implemented in different health systems with differing regulations, healthcare practices, requirements and regional conditions. A general overview of conceptual principles and characteristics is, therefore, challenging. Nonetheless, the results broaden the knowledge about the diversity of conceptual approaches, reveal common elements and summarise findings and knowledge gaps. This first-ever overview can, therefore, guide the future development of needs-based healthcare for tracheotomised patients with or without mechanical ventilation. The results highlight implications for future research and contribute to stimulating the international discourse on appropriate healthcare approaches healthcare, the value of existing concepts, their elements and interventions for the patient group.

## Conclusions

Concepts addressing the complex needs of long-term tracheotomised patients with or without mechanical ventilation aim to provide needs-based healthcare across sectors and settings. This scoping review points out their structural, organisational and processual key elements that represent elementary principles of comprehensive, integrated and need-based healthcare. They should, therefore, be carefully considered for future conceptual developments and discourses in Germany and elsewhere. Yet, the review also revealed considerable heterogeneity and lacking scientific knowledge as to whether and to what extent healthcare improvements can be achieved. Thus, conclusions on how to best respond to existing challenges to appropriate healthcare are hard to draw. Future research should, therefore, particularly describe and examine conceptual key elements, their impact on healthcare processes and outcomes, and their feasibility and replicability to other contexts. Approaches should also be systematically and comprehensively reported to identify and diffuse best practice. This would decisively support the further development of reasonable approaches to needs-based healthcare for the patient group.

## Additional Files

The additional files for this article can be found as follows:

10.5334/ijic.5429.s1 Appendix 1. References included in the Scoping Review.

10.5334/ijic.5429.s2 Appendix 2. Table 4: Evaluation characteristics and results.
