# Supplementary material for: Long-Term Care for Tracheotomised Patients With or Without Invasive Ventilation. Lessons Learned from a Scoping Review of International Concepts
Source: Int J Integr Care. 2020 Jul 17;20(3):3. doi: 10.5334/ijic.5429 (PMC7366865; doi:10.5334/ijic.5429)
Supplement: Appendix 1. — References included in the Scoping Review. [file ijic-20-3-5429-s1.pdf]

Appendix 1

References included in the Scoping Review

47. The Royal Children's Hospital Melbourne. Complex Care Hub. Coordination of care for children with chronic and complex medical needs. 2017 [cited 2019 Jun 20]. Available from: https://www.rch.org.au/uploadedFiles/Main/Pages/complex-care-hub/resources/Complex%20Care%20Hub%20brochure%20final.pdf.

48. The Royal Children's Hospital Melbourne. About Complex Care Hub. n.d. [cited 2019 Jun 19]. Available from: https://www.rch.org.au/complex-care-hub/.

49. McKinlay, L. Connected Care. Care Coordination for Children with Complex Healthcare Needs. In: Royal Australasian College of Physicians (RACP) Congress 2016 – Evolve Educate Engage. Adelaide, Australia, 2016 [cited 2019 Jun 20]. Available from: https://www.racp.edu.au/docs/default-source/Events/congress-2016-presentations/racp-16-monday-dr-lynne-mckinlay.pdf?sfvrsn=2%20

50. Children's Health Queensland Hospital and Health Service. Connected care and nurse navigator programs. 2017 [cited 2019 Jun 15]; Available from: https://www.childrens.health.qld.gov.au/lcch/patients-families/connected-care/.

51. Moss PW, Thompson S, Ehmer M, McKinlay L. The Connected Care Program – changing the way we care for Queensland's most vulnerable children with acute healthcare needs. Int J Integr Care 2018;18(1(S1)):73. DOI: http://doi.org/10.5334/ijic.s1073.

52. Duncan A. Victorian Respiratory Support Service. Ventilator-Assisted Living 2011;25(4):3. [cited 2019 May 20] Available from: https://docs.wixstatic.com/ugd/fef361_878252a35dbf4bf1abfb73b2ff851347.pdf.

53. Hannan LM, Sahi H, Road JD, McDonald CF, Berlowitz DJ, Howard ME. Care Practices and Health-related Quality of Life for Individuals Receiving Assisted Ventilation. A Cross-National Study. Ann Am Thorac Soc 2016;13(6):894-903. DOI: https://doi.org/10.1513/AnnalsATS.201509-590OC.

54. Austin Health Victoria. Departments. Victorian Respiratory Support Service. 2017 [cited 2019 Apr 24]. Available from: http://www.austin.org.au/vrss.

55. Tracheostomy Review and Management Service. TRAMS: Excellence in Tracheostomy Care. 2013 [cited 2019 May 13]. Available from: http://tracheostomyteam.org/.

56. Cameron TS, McKinstry A, Burt SK, Howard ME, Bellomo R, Brown D J, et al. Outcomes of patients with spinal cord injury before and after introduction of an interdisciplinary tracheostomy team. Crit Care Resusc 2009;11(1):14-9.

57. Cameron T, Duncan A. Austin Health Tracheostomy Clinical Policy. Management of Patients with Tracheostomy at Austin Health. Heidelberg, Victoria: Tracheostomy Policy and Procedure Review Committee, Austin Health, 2014 [cited 2019 May 23]. Available from: http://tracheostomyteam.org/data/uploads/pdf/overarching-policysent-to-ns-2-ext-website.pdf.

58. Cameron T, Warrillow S. Tracheostomy Management in the Antipodes. 2014 [cited 2019 May 23]. Available from: http://globaltrach.org/wp-content/uploads/2014/04/4.-The-State-of-Things-Australian-Perspective-Warrillow-Cameron.pdf.

59. Loeffler E, Rhida S, Cook-Major N. A partnership model for children with complex medical conditions: The Champlain Complex Care Programme in Canada. In: Governance International (eds.). Case Studies. Birmingham: Governance International. 2013 [cited 2019 Jun 06]. Available from: http://www.govint.org/good-practice/case-studies/a-partnership-model-for-children-with-complex-medical-conditions/.

60. Children's Hospital of Eastern Ontario. Champlain Complex Care Program. 2019 [cited 2019 Jun 06]. Available from: https://www.cheo.on.ca/en/clinics-services-programs/complex-care-program.aspx#Partner-organizations.

61. Marabelli M, Newell S, Gogan J. Pilot-testing a pediatric complex care coordination service. J Info Technol Teach Cases 2016;6(1):45-55. DOI: https://doi.org/10.1057/jittc.2015.14.

62. Haddad M. Value and Affordability in Paediatrics. Ensuring Equitable Care in Paediatrics in the Toronto Central Local Health Integration Network (TC LHIN). Toronto: Hospital of Sick Children, 2009 [cited 2019 May 23]. Available from: https://www.sickkids.ca/Hospital-Utilities/22854-Value-Affordability-Paediatrics-Report.pdf.

63. Cohen E, Friedman JN, Mahant S, Adams S, Jovcevska V, Rosenbaum P. The impact of a complex care clinic in a children's hospital. Child Care Health Dev 2010;36(4):574-82. DOI: https://doi.org/10.1111/j.1365-2214.2009.01069.x.

64. Cohen E, Lacombe-Duncan A, Spalding K, MacInnis J, Nicholas D, Narayanan UG, et al. Integrated complex care coordination for children with medical complexity: a mixed-methods evaluation of tertiary care-community collaboration. BMC Health Serv Res 2012;12(1):366. DOI: https://doi.org/10.1186/1472-6963-12-366.

65. Cohen E, Friedman JN. Caring for Children with Medical Complexity: Definitions, Challenges and Solutions. Curr Pediatr Rev 2012;8(2):93-102. DOI: https://doi.org/10.2174/157339612800681253.

66. Adams S, Cohen E, Mahant S, Friedman JN, MacCulloch R, Nicholas DB. Exploring the usefulness of comprehensive care plans for children with medical complexity (CMC): a qualitative study. BMC Pediatrics 2013;13:10. DOI: https://doi.org/10.1186/1471-2431-13-10.

67. The Hospital for Sick Children. Complex Care Program. 2014 [cited 2019 May 30]. Available from: http://www.sickkids.ca/PaediatricMedicine/What-we-do/Complex-Care-Program/Index.html.

68. Kingsnorth S, Lacombe-Duncan A, Keilty K, Bruce-Barrett C, Cohen E. Inter-organizational partnership for children with medical complexity: the integrated complex care model. Child Care Health Dev 2015;41(1):57-66. DOI: https://doi.org/10.12927/hcq.0000.22580.

69. Sperling C. Care Model – Tracheostomy & Ventilated Pediatric Patients Receiving Treatment in the Ambulatory Unit. Can J Crit Care Nurs 2017;28(2):64.

70. McKim DA. An External Review of the Provincial Respiratory Outreach Program. Life-giving mechanical ventilation for people with disabilities living in the community. Vancouver: BC Association of Individualized Technology and Supports for People with Disabilities Provincial Respiratory Outreach Program. 2009 [cited 2019 Jun 21]. Available from: http://bcits.org/wp-content/uploads/2016/05/BCITSReport_PROPBusPlanVers-Jan09Email.pdf.

71. BC Association for Individualized Technology and Supports for People with Disabilities. PROP Program. 2016 [cited 2019 May 18]. Available from: http://bcits.org/provincial-respiratory-outreach-program-prop/.

72. BC Association for Individualized Technology and Supports for People with Disabilities. Provincial Respiratory Outreach Program (PROP). Discharge Planning Guide. n.d. [cited 2019 May 21]; Available from: http://bcits.org/wp-content/uploads/2016/08/PROPDischargePlanningGuide.pdf.

73. McGill University Health Centre. Quebec National Program for Home Ventilatory Assistance. 2018 [cited 2019 May 16]. Available from: http://www.nphva.ca/.

74. National Program for Home Ventilatory Assistance. NPHVA platform. 2018 [cited 2019 May 23]. Available from: https://pnavd.net/?locale=en.

75. Project Steering Committee. A Systems Model to meet the health and supportive care needs of Adults living with Chronic Mechanical Ventilation in the South West LHIN. A Commitment to Excellence and Partnerships. 2013 [cited 2019 May 24]. Available from: http://www.southwestlhin.on.ca/~/media/sites/sw/PDF/MechVent/Final%20CMV%20Implementation%20Planning%20Report%20approved%20June%2027%202014.pdf?la=en.

76. Leasa D, Elson S. Building a Comprehensive System of Services to Support Adults Living with Long-Term Mechanical Ventilation. Can Respir J 2016:3185389. doi:10.1155/2016/3185389. DOI: https://doi.org/10.1155/2016/3185389

77. Moss C. Pilot project helps transition ventilated patients from ICU to their home communities. LHINfo Minute. Health Care Update. 2011 [cited 201 Jun 26]; Available from: http://www.centrallhin.on.ca/newsandevents/~/media/sites/central/uploadedfiles/Public_Community/News_Room/LHINfo_Minute/WestPark_LHINfoMinute_August2011_final.pdf.

78. West Park Health Care Centre. Transforming the Health System for the Care of Long-Term Ventilated Individuals, In: Canadian Foundation for Healthcare Improvement. National Health Leadership Conference, Ottawa, Canada, 2016, Jun 26. Ottawa: Canadian Foundation for Healthcare Improvement. 2016 [cited 2019 Jun 20]. Available from: http://www.nhlc-cnls.ca/wp-content/uploads/2016/11/Malek_Transforming-the-health1.pdf.

79. West Park Healthcare Centre. Long-Term Ventilation Centre of Excellence. 2016 [cited 2019 Jun 20]. Available from: http://ltvcoe.com/.

80. West Park Health Care Centre. Transitional Home Ventilation. Providing rehabilitation, training and education. n.d. [cited 2019 Jun 20]; Available from: https://www.westpark.org/en/Services/TransitionalHomeVentilation.

81. Ricart Campos S, Lasheras Soria E, Aldemira Liz A. PRINCEP program: clinical program for specialized and integrated care of paediatric patients with complex chronic conditions. Int J Integr Care 2016;16(6):1-2. DOI: http://doi.org/10.5334/ijic.2669.

82. Stuart M, Weinrich M. Integrated health system for chronic disease management: lessons learned from France. Chest 2004;125(2):695-703. DOI: https://doi.org/10.1378/chest.125.2.695.

83. Veale D. Chronic respiratory care and rehabilitation in France. Chron Resp Dis 2006;3(4):215-16. DOI: https://doi.org/10.1177/1479972306070070.

84. Fédération Antadir. [National Association for Homecare, Innovations and Research]. 2011 [cited 2019 Jul 26]. Available from: https://www.antadir.com/. [French].

85. University of Michigan Health System. Welcome to the Adult Assisted Ventilation Clinic. 2013 [cited 2018 Jul 14]. Available from: http://www.med.umich.edu/1libr/PedHomeVent/VentTransitionBook.pdf.

86. University of Michigan Health System. Eligibility for Care in the University of Michigan Assisted Ventilation Clinic (Adult Home Ventilatory Support Program). 2014 [cited 2019 Jul 15]; Available from: https://medicine.umich.edu/sites/default/files/content/downloads/ELIGIBILITY%20FOR%20CARE%20IN%20THE%20UNIVERISTY%20OF%20MICHIGAN%20ASSISTED%20VENTILATION%20CLINIC.pdf.

87. Brown J, Hanley J, Rochefort K, Kurili A, Schotland H, Sitrin RG. Multi-disciplinary Outpatient Care of Ventilator-Assisted Adults in a University Setting, Am J Respir Crit Care Med 2017;(195):A2339. [cited 2019 Jun 20] Available from: https://www.atsjournals.org/doi/abs/10.1164/ajrccm-conference.2017.195.1_MeetingAbstracts.A2339.

88. Michigan Medicine – University of Michigan. Assisted Ventilation Clinic. 2018 [cited 2019 Jun 15]. Available from: https://www.uofmhealth.org/conditions-treatments/pulmonary/assisted-ventilation-clinic.

89. Steiner BD, Denham AC, Ashkin E, Newton WP, Wroth T, Dobson LA. Community Care of North Carolina: Improving Care Through Community Health Networks. Ann Fam Med 2008;6(4):361-67. DOI: https://doi.org/10.1370/afm.866.

90. Stiles AD, Tayloe DT, Wegner SE. Comanagement of medically complex children by subspecialists, generalists, and care coordinators. Pediatrics 2014;134(2):203-5. DOI: https://doi.org/10.1542/peds.2013-3257

91. Community Care of North Carolina. Health Care Collaboration to Improve Care for North Carolina's Most Fragile Children. 2019 [cited 2019 Jun 19]. Available from: https://www.communitycarenc.org/newsroom/health-care-collaboration-improve-care-north-carolinas-most-fragile-children.

92. Community Care of North Carolina. Child Health Accountable Care Initiative of NC. 2019 [cited 2019 Sep 04]. Available from: http://www.npiedmontcc.org/programs-initiatives/health-initiatives/chacc-nc.

93. Petitgout JM, Pelzer DE, McConkey SA, Hanrahan K. Development of a hospital-based care coordination program for children with special health care needs. J Pediatr Health Care 2013;27(6):419-25. DOI: https://doi.org/10.1016/j.pedhc.2012.03.005.

94. University of Iowa Stead Family Children's Hospital. Continuity of Care. 2019 [cited 2019 Sep 04]. Available from: https://uichildrens.org/medical-services/continuity-care.

95. Petitgout JM. The Financial Impact of a Hospital-Based Care Coordination Program for Children with Special Health Care Needs. J Pediatr Health Care 2018;32(1):3-9. DOI: https://doi.org/10.1016/j.pedhc.2017.06.003.

96. C.S. Mott Children's Hospital Michigan Medicine. Home Ventilator Program. 2019 [cited 2019 Sep 04]. Available from: https://www.mottchildren.org/conditions-treatments/ped-home-vent.

97. Children's Hospital of Pittsburgh. Technology Assisted Children's Home Program. 2019 [cited 2019 May 29]. Available from: http://www.chp.edu/our-services/rehab-medicine/tachp.

98. Tamasitis J, Shesser L. A Hospital-to-Home Program for Ventilator-dependent Children Sets the Standard of Care. AARC Times 2012;36(10):44-52.

99. Children's Hospital of Philadelphia. Children's Hospital Home Care. 2018 [cited 2019 Jun 28]. Available from: https://www.chop.edu/centers-programs/childrens-hospital-home-care.

100. Gordon JB, Colby HH, Bartelt T, Jablonski D, Krauthoefer ML, Havens P. A tertiary care-primary care partnership model for medically complex and fragile children and youth with special health care needs. Arch Pediatr Adolesc Med 2007;161(10):937-44. DOI: https://doi.org/10.1001/archpedi.161.10.937.

101. Children's Hospital of Wisconsin. Special Needs Program. 2018 [cited 2018 Jun 06]. Available from: https://www.chw.org/medical-care/special-needs-services.

102. Children's Hospital of Wisconsin Tracheostomy and Home Ventilator Program. 2018 [cited 2019 Jun 06]. Available from: https://www.chw.org/medical-care/tracheostomy-home-ventilator.

103. Graham, RJ, Rodday AM, Parsons SK. Family-centered Assessment and Function for Children With Chronic Mechanical Respiratory Support. J Pediatr Health Care 2014;28(4):295-304. DOI: https://doi.org/10.1016/j.pedhc.2013.06.006.

104. Casavant DW, McManus ML, Parsons SK, Zurakowski D, Graham RJ. Trial of telemedicine for patients on home ventilator support: feasibility, confidence in clinical management and use in medical decision-making. J Telemed Telecare 2014;20(8):441-9. DOI: https://doi.org/10.1177/1357633X14555620.

105. Graham RJ, McManus ML, Rodday AM, Weidner RA, Parsons SK. Chronic respiratory failure: utilization of a pediatric specialty integrated care program. Healthcare 2017;5(1):23-8. DOI: https://doi.org/10.1016/j.hjdsi.2016.04.002.

106. Boston Children's Hospital Critical Care, Anesthesia, Perioperative, Extension (C.A.P.E.) and Home Ventilation Program. 2019 [cited 2019 Sep 04]. Available from: http://www.childrenshospital.org/centers-and-services/programs/a-_-e/critical-care-anesthesia-perioperative-extension-and-home-ventilation-program.

107. Bunch D. There's No Place Like Home. AARC Times 2016;40(8):38-41.

108. Palfrey JS, Sofis LA, Davidson EJ, Liu J, Freeman L, Ganz ML. The Pediatric Alliance for Coordinated Care: evaluation of a medical home model. Pediatrics 2004;113(5 Suppl):1507-16.

109. Boston Children's Hospital Complex Care Service Program. 2019 [cited 2019 Sep 04]. Available from: http://www.childrenshospital.org/centers-and-services/programs/a-_-e/complex-care-service-program.

110. Cristea AI, Carroll AE, Davis SD, Swigonski NL, Ackerman VL. Outcomes of children with severe bronchopulmonary dysplasia who were ventilator dependent at home. Pediatrics 2013;132(3):e727-34. DOI: https://doi.org/10.1542/peds.2012-2990.

111. Riley Hospital for Children at Indiana University Health. Home Ventilation Program. 2019 [cited 2019 Sep 04]. Available from: https://www.rileychildrens.org/departments/home-ventilation-program.

112. Pandey SK, Mussman MG, Moore HW, Folkemer JG, Kaelin JJ. An assessment of Maryland Medicaid's Rare and Expensive Case Management Program. Eval Health Prof 2000;23(4):457-79. DOI: https://doi.org/10.1177/01632780022034723.

113. State of Maryland. Code of Maryland Regulations. Title 10. Department of Health & Mental Hygiene. Part 2. Subtitle 09. Medical care programs. Chapter 10.09.69. Maryland Medicaid Managed Care Program: Rare and Expensive Case Management. 2016 [cited 2019 May 25]. Available from: http://mdrules.elaws.us/comar/10.09.69.

114. Kelly A, Golnik A, Cady R. A Medical Home Center: Specializing in the Care of Children with Special Health Care Needs of High Intensity. Matern Child Health J 2008;12(5):633-40. DOI: https://doi.org/10.1007/s10995-007-0271-7.

115. Cady R, Kelly A, Finkelstein S. Home telehealth for children with special health-care needs. J Telemed Telecare 2008;14(4):173-7. DOI: https://doi.org/10.1258/jtt.2008.008042.

116. Cady R, Finkelstein S, Kelly A. A telehealth nursing intervention reduces hospitalizations in children with complex health conditions. J Telemed Telecare 2009;15(6):317-20. DOI: https://doi.org/10.1258/jtt.2009.090105.

117. Looman WS, Erickson MM, Garwick AW, Cady R, Kelly A, Pettey C, et al. Meaningful Use of Data in Care Coordination by the Advanced Practice Registered Nurse: The TeleFamilies Project. Cin-Comput Inform Nu 2012;30(12):649-54. DOI: https://doi.org/10.1097/NXN.0b013e318266caf2.

118. Looman WS, Presler E, Erickson MM, Garwick AW, Cady R, Kelly AM, et al. Care Coordination for Children with Complex Special Health Care Needs: The Value of the Advanced Practice Nurse's Enhanced Scope of Knowledge and Practice. J Pediatr Health Care 2013;27(4):293-303. DOI: https://doi.org/10.1016/j.pedhc.2012.03.002.

119. Cady R, Kelly A, Finkelstein S, Looman WS, Garwick AW. Attributes of advanced practice registered nurse care coordination for children with medical complexity. J Pediatr Health Care 2014;28(4):305-12. DOI: https://doi.org/10.1016/j.pedhc.2013.06.005.

120. Cady R, Looman W, Lindeke L, LaPlante B, Lundeen B, Seeley A, et al. Pediatric Care Coordination: Lessons Learned and Future Priorities. Online J Issues Nurs 2015;20(3):3. DOI: 10.3912/OJIN.Vol20No03Man03.

121. Cady R, Erickson M, Lunos S, Finkelstein SM, Stanley M, Looman W, et al. Meeting the Needs of Children with Medical Complexity Using a Telehealth Advanced Practice Registered Nurse Care Coordination Model. Matern Child Health J 2015;19(7):1497-506. DOI: https://doi.org/10.1007/s10995-014-1654-1.

122. Looman WS, Antolick M, Cady R, Lunos S, Garwick AE, Finkelstein S. Effects of a Telehealth Care Coordination Intervention on Perceptions of Health Care by Caregivers of Children with Medical Complexity: A Randomized Controlled Trial. J Pediatr Health Care 2015;29(4):352-63. DOI: https://doi.org/10.1016/j.pedhc.2015.01.007.

123. Richardson J, Khan MA, Chen G, Iezzi A, Meaxwell A. Population Norms and Australian Profile using the Assessment of Quality of Life (AQoL)8D Utility Instrument. Research Paper 2012 (72). 2012 [cited 2019 May 22]. Available from: https://business.monash.edu/__data/assets/pdf_file/0006/896442/researchpaper72.pdf.

124. Windisch W, Freidel K, Schucher B, Baumann H, Wiebel M, Matthys H, et al. The Severe Respiratory Insufficiency (SRI) Questionnaire: a specific measure of health-related quality of life in patients receiving home mechanical ventilation. J Clin Epidemiol 2003;56(8):752-9. DOI: https://doi.org/10.1016/S0895-4356(03)00088-X.

125. Katz S, Ford AB, Moskowitz RW, Jackson BA, Jaffe MW. Studies of illness in the aged. The index of ADL: a standardized measure of biological and psychological function. JAMA 1963;185:914-9. DOI: https://doi.org/10.1001/jama.1963.03060120024016.

126. Praed Foundation. Child and adolescent needs and strengths. Coordination of Complex Care Program (CANS-CCC). Children/Youth 7 – 17 years old. Manual. 2013 [cited 2019 May 24]. Available from: https://praedfoundation.org/general-manuals-cans/?b5-file=1434&b5-folder=1405.

127. Ware JE, Gandek B. The SF-36 Health Survey: Development and Use in Mental Health Research and the IQOLA Project. Int J Ment Health 1994;23(2):49-73. DOI: https://doi.org/10.1080/00207411.1994.11449283.

128. King SM, Rosenbaum PL, King GA. Parents’ perceptions of caregiving: development and validation of a measure of processes. Dev Med Child Neurol 1996;38(9):757-72. DOI: https://doi.org/10.1111/j.1469-8749.1996.tb15110.x.

129. Larsen DL, Attkisson CC, Hargreaves WA, Nguyen TD. Assessment of client/patient satisfaction: development of a general scale. Eval Program Plann 1979;2(3):197-207. DOI: https://doi.org/10.1016/0149-7189(79)90094-6.

130. Varni JW, Seid M, Kurtin PS. PedsQL 4.0: reliability and validity of the Pediatric Quality of Life Inventory version 4.0 generic core scales in healthy and patient populations. Med Care 2001;39(8):800-12. DOI: https://doi.org/10.1097/00005650-200108000-00006.

131. Narayanan UG, Fehlings D, Weir S, Knights S, Kiran S, Campbell K. Initial development and validation of the Caregiver Priorities and Child Health Index of Life with Disabilities (CPCHILD). Dev Med Child Neurol 2006;48(10):804-12. DOI: https://doi.org/10.1017/S0012162206001745.

132. Antonelli RC, Stille CJ, Antonelli DM. Care coordination for children and youth with special health care needs: a descriptive, multisite study of activities, personnel costs, and outcomes. Pediatrics 2008;122(1):e209-16. DOI: https://doi.org/10.1542/peds.2007-2254.

133. Goldstein E, Cleary PD, Langwell KM, Zaslavsky AM, Heller A. Medicare Managed Care CAHPS®: A Tool for Performance Improvement. Health Care Financ Rev 2001;22(3):101-7.

134. National Center for Health Statistics. 2005-06 National Survey of Children with Special Health Care Needs. Questionaire (Englisch). 2008 [cited 2019 May 24]. Available from: https://www.cdc.gov/nchs/data/slaits/NSCSHCNIIEnglishQuest.pdf.

135. Dyer N, Sorra JS, Smith SA, Cleary PD, Hays RD. Psychometric properties of the Consumer Assessment of Healthcare Providers and Systems (CAHPS®) Clinician and Group Adult Visit Survey. Med Care 2012;50(Suppl):S28-S34. DOI: https://doi.org/10.1097/MLR.0b013e31826cbc0d.

136. Looman WS. Development and testing of the social capital scale for families of children with special health care needs. Res Nurs Health 2006;29(4):325-36. DOI: https://doi.org/10.1002/nur.20148.
