# Supplementary figures and images for: Long-Term Care for Tracheotomised Patients With or Without Invasive Ventilation. Lessons Learned from a Scoping Review of International Concepts
Source: Int J Integr Care. 2020 Jul 17;20(3):3. doi: 10.5334/ijic.5429 (PMC7366865; doi:10.5334/ijic.5429)

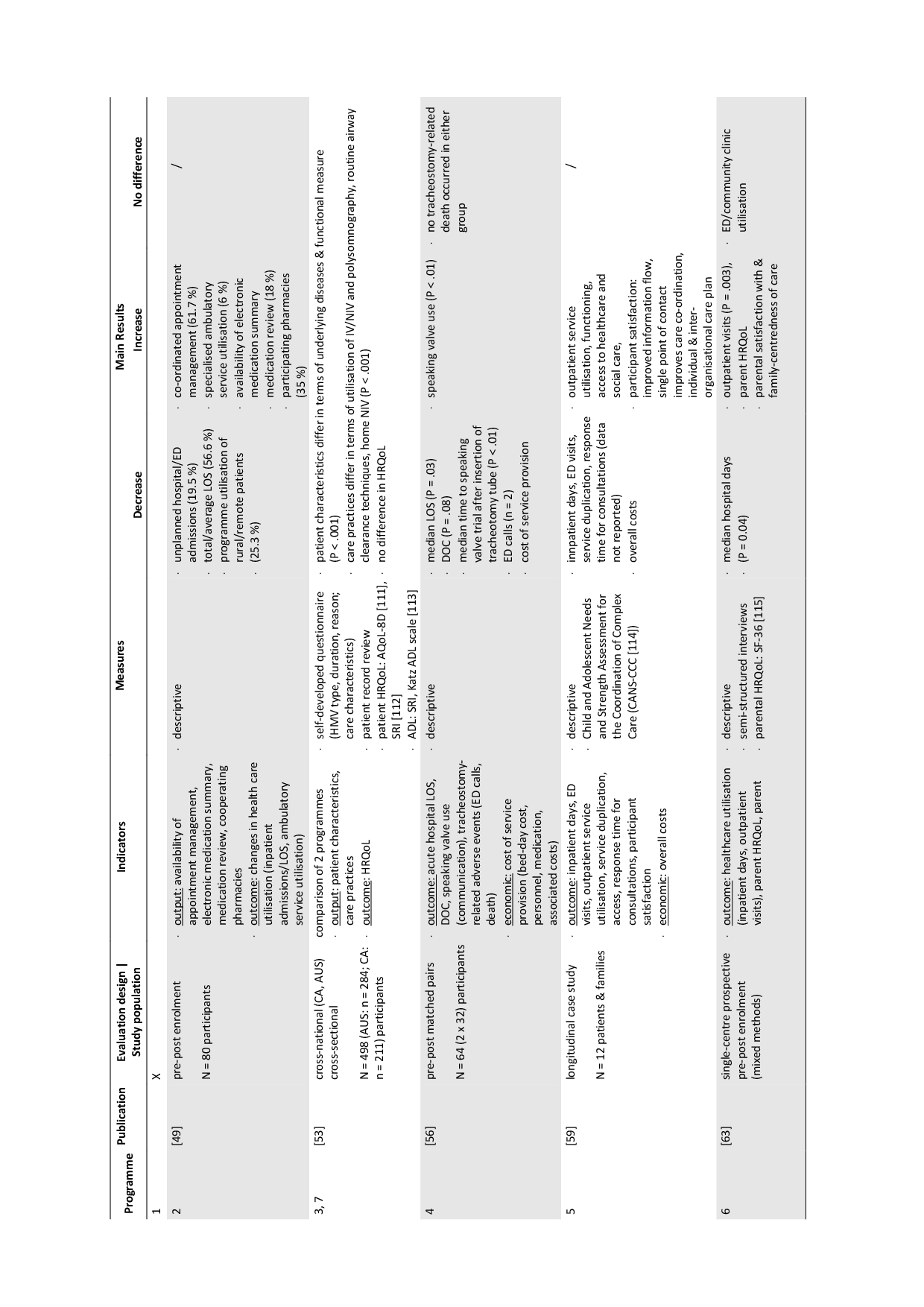

Supplement: Appendix 2. — Table 4: Evaluation characteristics and results. [file ijic-20-3-5429-s2.zip › 200506_RT1983_table4_evaluation-001.png]

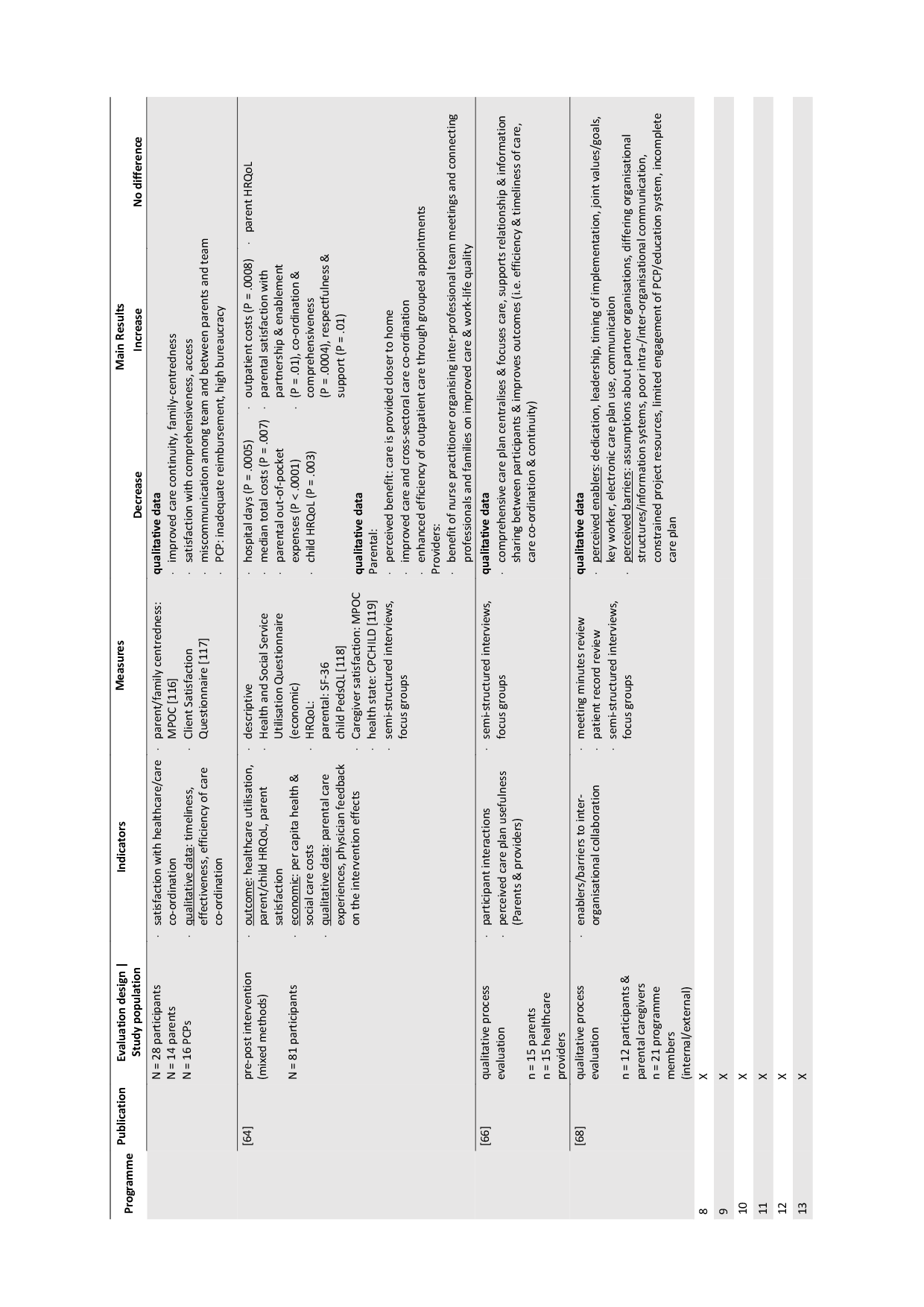

Supplement: Appendix 2. — Table 4: Evaluation characteristics and results. [file ijic-20-3-5429-s2.zip › 200506_RT1983_table4_evaluation-002.png]

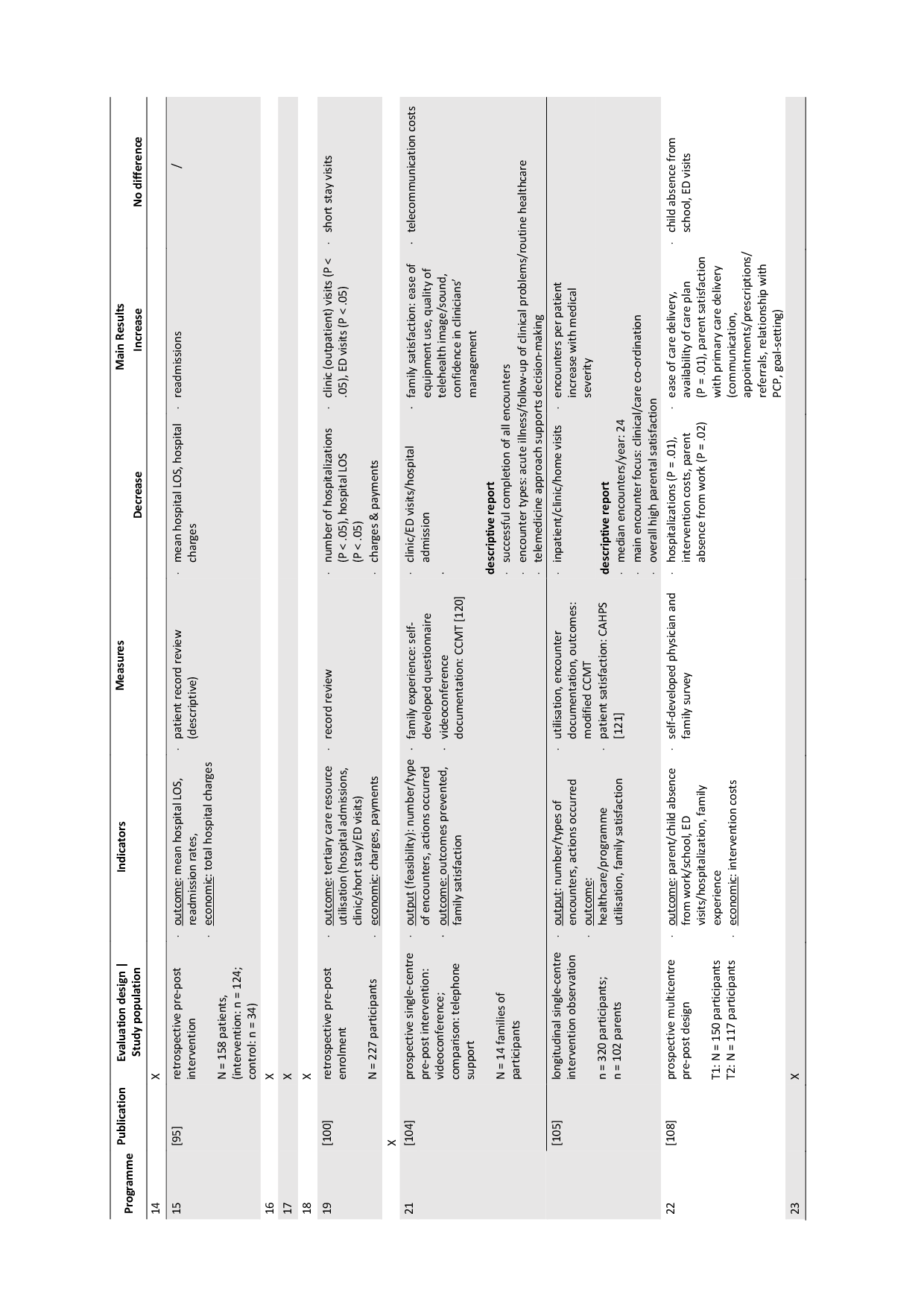

Supplement: Appendix 2. — Table 4: Evaluation characteristics and results. [file ijic-20-3-5429-s2.zip › 200506_RT1983_table4_evaluation-003.png]

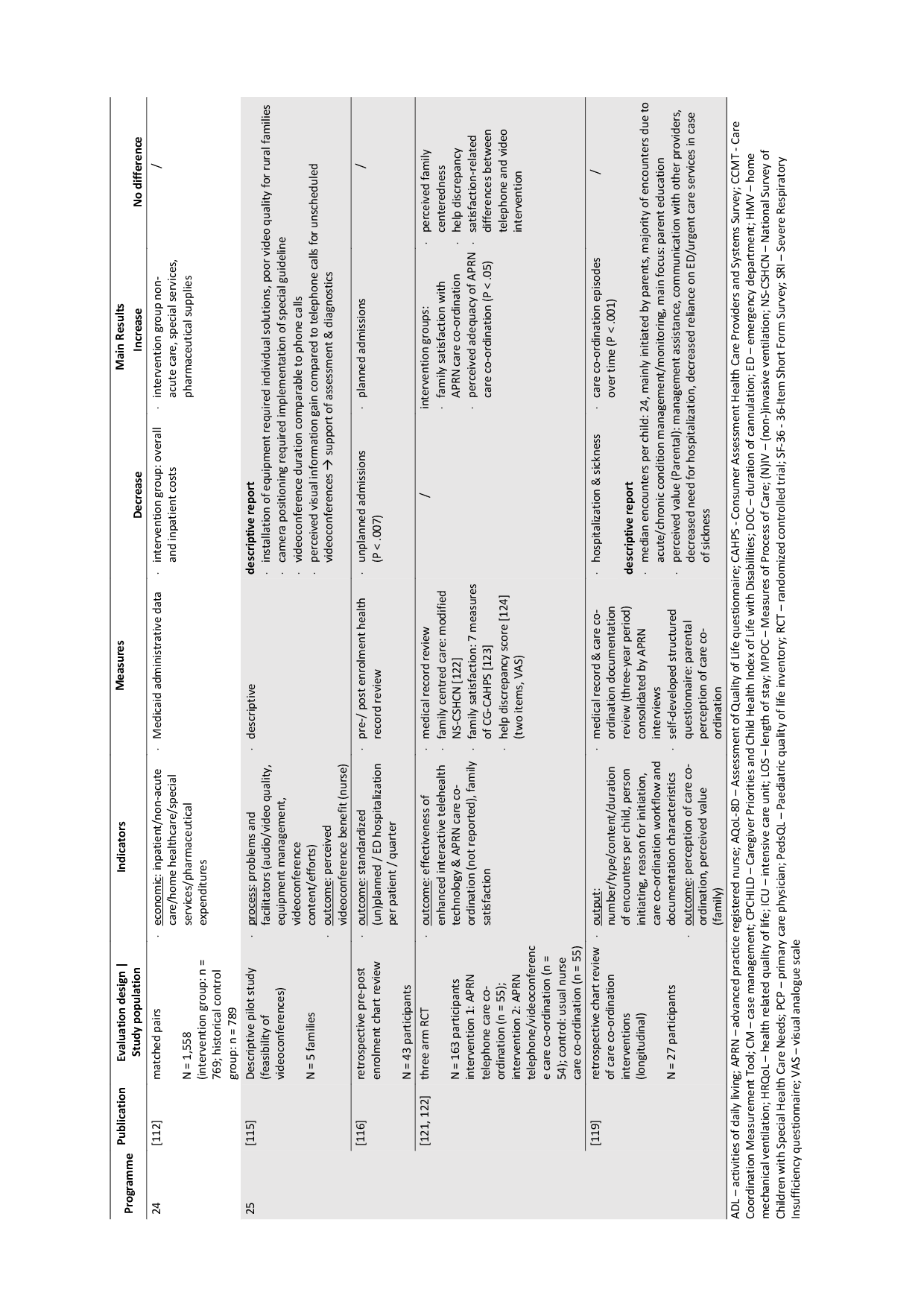

Supplement: Appendix 2. — Table 4: Evaluation characteristics and results. [file ijic-20-3-5429-s2.zip › 200506_RT1983_table4_evaluation-004.png]
